# Potential Synergistic Effect between Niraparib and Statins in Ovarian Cancer Clinical Trials

**DOI:** 10.1158/2767-9764.CRC-24-0191

**Published:** 2025-01-29

**Authors:** Hailei Zhang, Anna Rutkowska, Antonio González-Martín, Mansoor R. Mirza, Bradley J. Monk, Ignace Vergote, Bhavana Pothuri, Whitney A. Spannuth Graybill, Carsten Goessel, Olena Barbash, Giovanna Bergamini, Bin Feng

**Affiliations:** 1 Precision Medicine, R&D, GSK, Waltham, Massachusetts.; 2 Cellzome, a GSK Company, R&D, GSK, Heidelberg, Germany.; 3 Medical Oncology Department, Clínica Universidad de Navarra, Madrid, Spain.; 4 Center for Applied Medical Research (CIMA), Pamplona, Spain.; 5 Grupo Español de Investigación en Cáncer de Ovario (GEICO), Madrid, Spain.; 6 Nordic Society of Gynaecological Oncology (NSGO) and Department of Oncology Rigshospitalet–Copenhagen University Hospital, Copenhagen, Denmark.; 7 Florida Cancer Specialists and Research Institute, West Palm Beach, Florida.; 8 Gynecologic Oncology, Katholieke Universiteit Leuven, Leuven, Belgium.; 9 Department of Obstetrics and Gynecology, NYU School of Medicine, New York City, New York.; 10 Department of Obstetrics and Gynecology, Medical University of South Carolina, Charleston, South Carolina.; 11 Clinical Development, Oncology, GSK, Waltham, Massachusetts.; 12 Precision Medicine, GSK, Collegeville, Pennsylvania, Philadelphia.

## Abstract

This study investigates the potential clinical synergy between the PARP inhibitor niraparib (Zejula) and concomitant statins, exploring their combined effects on progression-free survival (PFS) in patients with ovarian cancer. We retrospectively analyzed niraparib registrational clinical trials in ovarian cancer to investigate potential interactions between niraparib and statins. In the PRIMA trial, patients receiving niraparib demonstrated improved PFS compared with those on placebo (HR = 0.62; *P* < 0.001; median PFS 13.8 vs. 8.2 months). The *post hoc* analysis revealed that patients receiving maintenance niraparib who reported concomitant use of statins exhibited significantly improved PFS compared with those on placebo with concomitant statins (HR = 0.34; *P* < 0.001; median PFS 18.2 vs. 6.0 months). Notably, the improved efficacy in the two-arm comparison of concomitant statin patients was much better than that in the two-arm comparison of those patients without statin, as reflected in the niraparib–statin interaction (*P* = 0.005). These findings suggest novel opportunities in oncology for the use of statins in combination therapies with PARP inhibitors and emphasize the need for further investigation.

**Significance:**

The presented retrospective analysis suggests, to the best of our knowledge for the first time, a potential significant interaction between statins and niraparib in clinical settings. Nevertheless, further investigations are required to gain a better understanding of the potential clinical benefit.

## Introduction

Ovarian cancer, a complex and often challenging malignancy, poses significant therapeutic dilemmas due to its propensity for late-stage diagnosis and inherent heterogeneity. In recent years, the advent of targeted therapies has, however, provided a beacon of hope in the quest for more effective and personalized interventions. Among these, PARP inhibitors (PARPi) have emerged as ground-breaking agents, showcasing significant promise in the management of ovarian cancer (1–4).

PARPis represent a cutting-edge approach to cancer treatment by exploiting specific vulnerabilities in cancer cells, particularly those associated with defects in DNA repair mechanisms (5). Ovarian cancer is notorious for harboring genetic alterations, and a significant subset of cases is characterized by mutations in genes such as *BRCA1* and *BRCA2*, integral players in the DNA repair pathway called homologous recombination repair. In this homologous recombination–deficiency (HRd) molecular landscape, PARPis have demonstrated exceptional efficacy, capitalizing on the concept of synthetic lethality (6). Among all PARPis, olaparib (7) and niraparib (8) are the only PARPis approved as first-line maintenance therapy for ovarian cancer, following response to platinum-based chemotherapy. Preclinical and clinical studies have demonstrated that tumor cells that are homologous recombination–proficient (HRp) may also respond to PARPis (8, 9).

Niraparib has demonstrated significant single-agent activity in ovarian cancer (8). However, the potential for enhanced outcomes lies in its strategic pairing with other agents, ranging from traditional chemotherapeutics to emerging immunomodulatory drugs (10). Among those, statins, the FDA-approved cholesterol-lowering drugs, potent inhibitors of the rate-limiting enzyme of the mevalonate pathway, 3-hydroxy-3-methylglutaryl-CoA reductase (HMGCR; ref. 11), have been reported to enhance the efficacy of conventional anticancer therapies (11–13). To explore potential benefits of such combination in case of PARPis, we investigated the effects of statins when concomitantly administered with niraparib to patients enrolled in niraparib ovarian clinical registration trials [PRIMA (first-line maintenance; ref. 8), NOVA (second-line and above maintenance; ref. 14), and QUADRA (late-line treatment; ref. 15)]. The statin–niraparib combination showed significantly better progression-free survival (PFS) efficacy compared with niraparib monotherapy in both PRIMA and QUADRA, but not in NOVA due to the limited number of patients in the statin subgroups. Furthermore, in the PRIMA clinical study, a significant interaction was observed between niraparib and concomitant statins, with the longest median PFS (mPFS) recorded for the subgroup of patients who received niraparib and concomitant statins, as opposed to the shortest mPFS being observed for patients who received placebo and concomitant statins. These findings suggest that simultaneous inhibition of the PARP activity and cholesterol biosynthesis pathways may provide therapeutic advantages in patients with ovarian cancer.

## Materials and Methods

### Statistical analysis

The statistical analysis for the niraparib statin interaction sub study was strictly exploratory in nature. To assess the potential synergistic combination effect between niraparib and statin, subgroup analyses were performed and niraparib treatment by statin concomitant interaction terms were tested in the Cox regression model. The stratified Cox model for interaction testing included three stratification factors for the PRIMA study: response to first platinum, homologous recombination deficiency (HRd) status, and neoadjuvant usage; an additional analysis was performed including type of statin, statin dose level, age, and weight as additional stratification factors; finally, niraparib starting dose level (200 or 300 mg) was added as one more stratification factor. For the NOVA study, the stratified Cox model for interaction testing included three stratification factors: the best response during the last platinum, the use of bevacizumab in conjunction with the penultimate or last platinum regimen, and time to progression after completion of the penultimate platinum regimen (6 to <12 months vs. ≥12 months). For the QUADRA study, the stratified Cox model for interaction testing included three stratification factors: line of therapy, best response to last platinum regimen, and the HRd status.

As the interaction test is acknowledged to have low power, a trend may be indicated by a *P* value marginally exceeding the 0.05 threshold. HRs were estimated with two-sided 95% confidence intervals (CI) using a stratified Cox proportional hazards model, with the stratification factors used in randomization.

### Data availability

GSK makes available anonymized individual participant data and associated documents from interventional clinical studies that evaluate medicines, upon approval of proposals submitted to www.clinicalstudydatarequest.com. To access data for other types of GSK sponsored research, for study documents without patient-level data, and for clinical studies not listed, please submit an enquiry via this website.

## Results

To explore potential clinical benefits of combination therapy with PARPis and cholesterol-lowering drugs, we investigated the effect of statins, HMGCR inhibitors, when administered as concomitant medication to patients with ovarian cancer enrolled in three niraparib monotherapy registrational clinical trials. The retrospective analysis revealed that the second-line maintenance NOVA (ENGOT-OV16; ref. 14) trial, leading to niraparib approval, had only 71 patients who took concomitant statins, which limited the assessment (Table 1; Supplementary Table S1). In contrast, both the forth-line treatment trial QUADRA (ECOG; ref. 15) and the first-line maintenance PRIMA (8) trial (ENGOT-OV26/GOG-3012) had more patients, *n* = 105 and *n* = 143, respectively (Table 1; Supplementary Table S1).

**Table 1:** Number of patients with and without statin concomitant in the PRIMA, NOVA, and QUADRA clinical trials

	Patients with statin concomitant		Patients without statin concomitant	
	Placebo	Niraparib	Placebo	Niraparib
PRIMA	51	92	193	392
NOVA	23	48	156	319
QUADRA	n.a.^a^	105	n.a.^a^	356

a A single-arm study, i.e., not randomized, no placebo patients.

### *Post hoc* analysis of the PRIMA clinical study to investigate interaction between niraparib and statins

In PRIMA (8), a randomized, double-blind, phase 3 trial, patients with newly diagnosed advanced ovarian cancer were randomly assigned in a 2:1 ratio to receive niraparib or placebo once daily after a response to platinum-based chemotherapy. In the overall population, mPFS was significantly longer in the niraparib group than that in the placebo group. Significant prolongation was also observed in the group of patients with HRp tumors, although the HRd group benefitted most from niraparib treatment (Supplementary Table S2).

**Figure 1:**
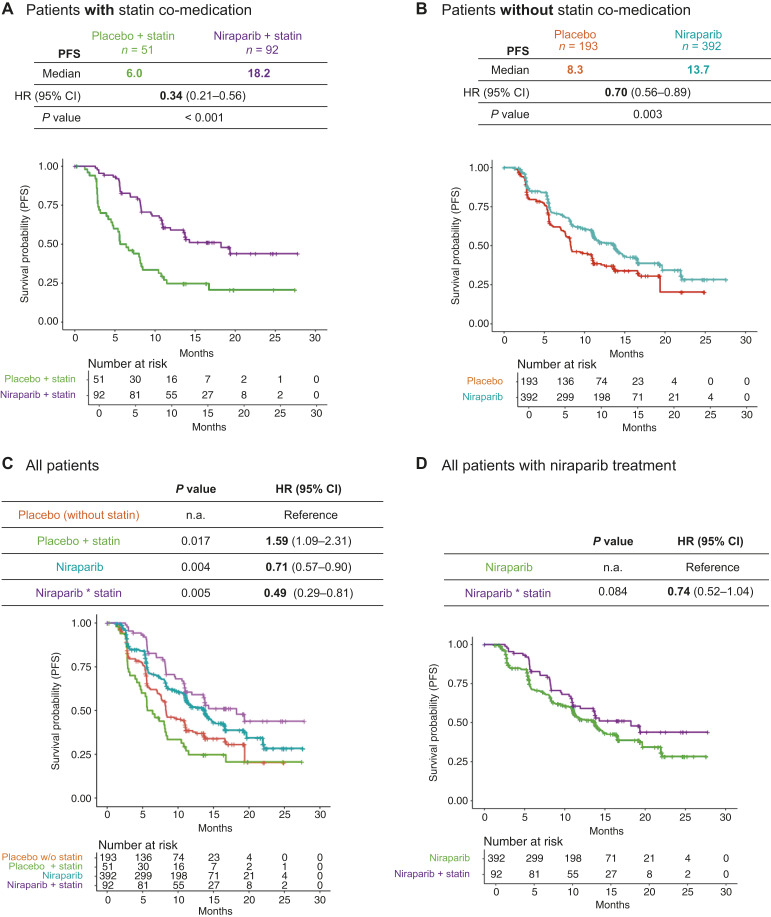
Retrospective analysis of PRIMA clinical trial–the interaction between niraparib and statin is significant. **A,** Kaplan–Meier estimation of PFS comparing patients treated with niraparib (*n* = 92) and placebo (*n* = 51) in the cohort with statin concomitant; stratified analysis with three randomization factors (response to first platinum, HRd status, and neoadjuvant usage); mPFS, HR, and log-rank test *P* value are reported. **B,** Kaplan–Meier estimation of PFS comparing patients treated with niraparib (*n* = 392) and placebo (*n* = 193) in the cohort without statin concomitant. **C,** Kaplan–Meier estimation of PFS comparing all four arms (the placebo without statins was the reference arm for comparison to the other three arms: niraparib, niraparib + statin, and placebo + statin). Statin and niraparib showed the synergistic combination effect (significant interaction) in this retrospective analysis. No significant differences in baseline characteristics (e.g., age and weight) across groups were observed (Supplementary Table S3). **D,** Kaplan–Meier estimation of PFS comparing niraparib to niraparib + statin arm.

Using the data published by Gonzalez-Martin and colleagues (8), the following groups were compared: (i) niraparib and statin group (*n* = 92); (ii) placebo and statin group (*n* = 51); (iii) niraparib group (*n* = 392), and (iv) placebo group (*n* = 193). Patients who received niraparib with concomitant statins had improved PFS compared with patients who received placebo with concomitant statins (HR = 0.34; 95% CI, 0.21–0.56; *P* < 0.001; Fig. 1A). The HR of those patients was much lower than for the group who did not take concomitant statins (0.34 vs. 0.7; Fig. 1A and B). Patients who received niraparib and concomitant statin had a longer mPFS than patients who received niraparib without concomitant statin (18.2 vs. 13.7 months; Fig. 1A and B). Importantly, the improved efficacy in the two-arm comparison of concomitant statin patients was much better than that in the two-arm comparison of those patients without statin, as reflected in the niraparib–statin interaction (*P* = 0.005, HR = 0.49, 95% CI, 0.29–0.81; Fig. 1C).

Patient characteristics and baseline demographics for all four groups were mostly balanced, except that age and weight were higher in the statin groups (Supplementary Table S3), which is not surprising for patients with high cholesterol levels and receiving statin treatment. In line with this observation, the HR for placebo with concomitant statins compared with the placebo-only group was 1.59 (95% CI, 1.09–2.31), showing the worst outcome across all the groups (Fig. 1C). Due to this significant difference between both placebo groups, the true effect of simultaneous modulation of PARP enzymatic activity and cholesterol biosynthesis is more accurately mirrored by the comparison of niraparib treatment to the respective placebo (Fig. 1A and B), or comparison of both arms to the same placebo (Fig. 1C) rather than niraparib to niraparib with concomitant statins (Fig. 1D).

An additional analysis accounting for patient characteristics (age and weight) and statin treatment (type of statin and its dose level) also indicated that niraparib and statin interaction is significant in this *post hoc* analysis of the PRIMA trial (HR = 0.31; 95% CI, 0.11–0.87; *P* = 0.026; Supplementary Fig. S1).

A similar analysis that included the different starting doses of niraparib used in the PRIMA trial (8) as an additional stratification factor excluded the possibility that the different niraparib doses could have invalidated the conclusions derived from the previous analysis (Supplementary Fig. S2).

Also, potential drug–drug interactions are unlikely to play a role in the clinical observation, as niraparib does not interact with the subfamily CYP3A of cytochrome P450 involved in drug metabolism of many statins as well as cholesterol, steroids, and other lipids (16, 17), and shows only marginal affinity for P-glycoprotein (IC_50_ = 161 μmol/L). We could also rule out adverse events (AE) as a potential confounding factor of the improved outcome observed for the group of patients receiving niraparib and concomitant statins, as incidence of AEs was not lower for this group (Supplementary Table S4). Of note, cholesterol levels were not monitored in this study.

**Figure 2:**
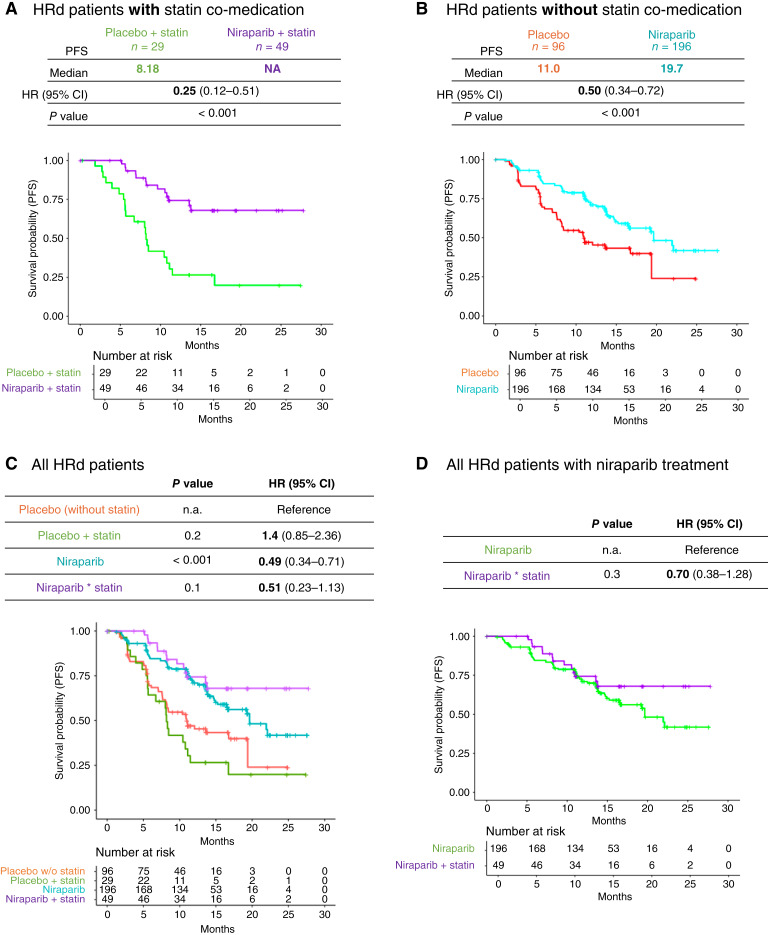
Retrospective analysis of the PRIMA clinical trial in the HRd group. **A,** Kaplan–Meier estimation of PFS comparing patients classified as HRd treated with niraparib (*n* = 49) and placebo (*n* = 29) in the cohort with statin concomitant; stratified analysis with three randomization factors (response to first platinum, HRd, status, and neoadjuvant usage); mPFS, HR, and log-rank test *P* value are reported. **B,** Kaplan–Meier estimation of PFS comparing patients treated with niraparib (*n* = 196) and placebo (*n* = 96) in the cohort without statin concomitant. **C,** Kaplan–Meier estimation of PFS comparing all four arms (the placebo without statins was the reference arm for comparison to the other three arms: niraparib, niraparib + statin, and placebo + statin). **D,** Kaplan–Meier estimation of PFS comparing niraparib to niraparib + statin arm.

**Figure 3:**
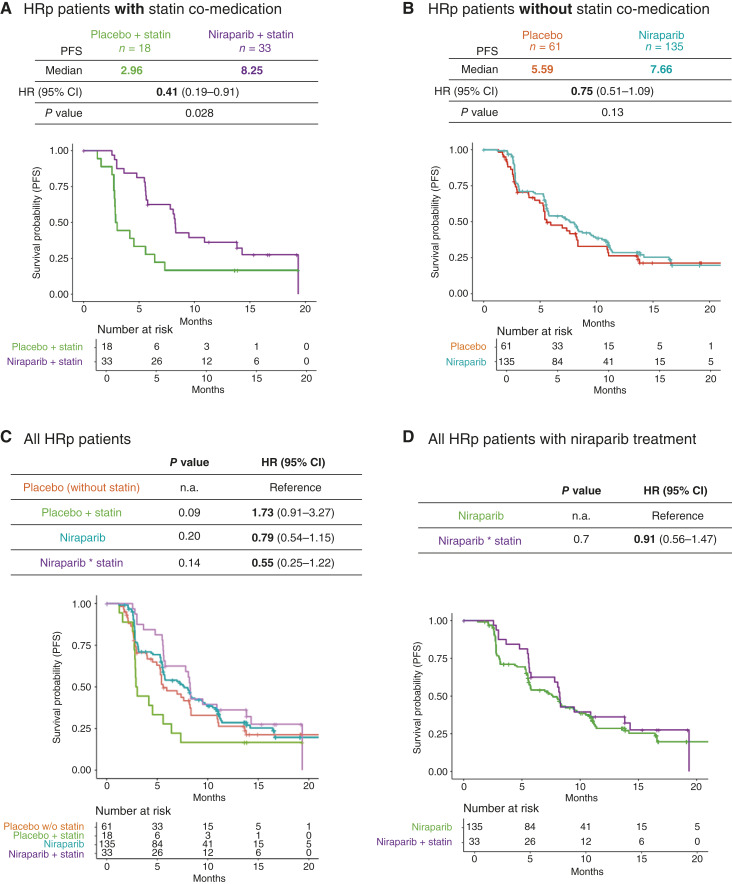
Retrospective analysis of PRIMA clinical trial in the HRp group. **A,** Kaplan–Meier estimation of PFS comparing patients classified as HRp treated with niraparib (*n* = 33) and placebo (*n* = 18) in the cohort with statin concomitant; stratified analysis with three randomization factors (response to first platinum, HRd status, and neoadjuvant usage); mPFS, HR, and log-rank test *P* value are reported. **B,** Kaplan–Meier estimation of PFS comparing patients treated with niraparib (*n* = 135) and placebo (*n* = 61) in the cohort without statin concomitant. **C,** Kaplan–Meier estimation of PFS comparing all four arms (the placebo without statins was the reference arm for comparison to the other three arms: niraparib, niraparib + statin, and placebo + statin). **D,** Kaplan–Meier estimation of PFS comparing niraparib to niraparib + statin arm.

To investigate the interaction of niraparib and statins on tumors with different HR status, we further divided PRIMA patients into HRd and HRp groups (Figs. 2 and 3). Within the HRd cohort, patients who received niraparib with concomitant statin treatment (*n* = 49) showed improved PFS compared with patients who received placebo and concomitant statins (*n* = 29; mPFS >30 vs. 8.18 months; HR = 0.25; 95% CI, 0.12–0.51; *P* < 0.001; Fig. 2A). Similarly, patients without concomitant statin also demonstrated improved mPFS with niraparib (*n* = 196) compared with placebo (*n* = 96; mPFS 19.6 vs. 11.0 months; HR = 0.50; 95% CI, 0.34–0.72; *P* < 0.001; Fig. 2B). In the HRp cohort, patients treated with niraparib who took concomitant statins (*n* = 33) had a superior outcome for PFS compared with the group treated with placebo who took concomitant statins (*n* = 18; mPFS 8.25 vs. 2.96 months; HR = 0.42; 95% CI, 0.19–0.91; *P* = 0.03; Fig. 3A). Among HRp patients not receiving concomitant statin, a trend toward improved mPFS was observed for the niraparib group (*n* = 135) compared with placebo (*n* = 61; mPFS 7.66 vs. 5.59 months; HR = 0.75; 95% CI, 0.51–1.09; *P* = 0.13; Fig. 3B). Overall, patients who received niraparib and concomitant statins exhibited longer PFS compared with those treated with niraparib who did not take statins, irrespective of the HR status of the treated tumors (Figs. 2C, D, 3C, and D). As mentioned above, due to the worse outcome of the placebo with concomitant statins arm as compared with the placebo-only group, the comparison of niraparib treatment to the respective placebo (Figs. 2A–C and 3A–C) more accurately reflected the effect of simultaneous modulation of PARP enzymatic activity and cholesterol biosynthesis. Finally, the inclusion of stratification factors covering patient characteristics (age and weight) and statin treatment (type of statin and dose level) also reported improved mPFS for patients treated with niraparib and concomitant statins (Supplementary Fig. S3).

### *Post hoc* analysis of the QUADRA clinical study to investigate interaction between niraparib and statins

**Figure 4:**
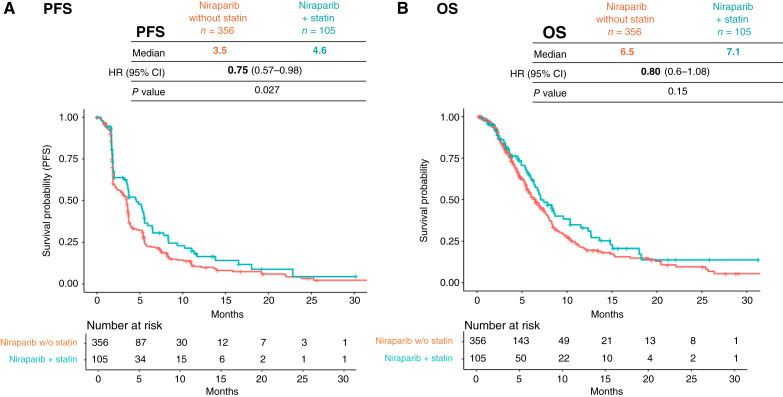
Retrospective analysis of the QUADRA clinical trial. **A,** Kaplan–Meier estimation of PFS comparing patients treated with niraparib in the cohort with statin concomitant (*n* = 105) and patients not receiving stains (*n* = 356); placebo arm was missing; a Cox proportional hazards model using line of therapy, response to last platinum regiment, and HRd status as stratification factors; mPFS, HR, and log-rank test *P* value are reported. **B,** Kaplan–Meier estimation of OS comparing patients as described in **A**. Statin–niraparib combination shows directionally better efficacy than niraparib. OS, overall survival.

In QUADRA (15), an open-label, single-arm, phase 2 registration study, patients with relapsed ovarian cancer were assigned to receive niraparib after three or more prior lines of chemotherapy. Unlike NOVA and PRIMA, the QUADRA trial did not have a placebo control arm. In the overall population, clinically relevant activity of niraparib was observed, especially in patients with HRd platinum-sensitive disease including *BRCA wild-type* disease (15). All patients were categorized based on concomitant statin use into two groups: (i) niraparib and concomitant statin group (*n* = 105) and (ii) niraparib-only group (*n* = 356). Patients treated with niraparib who took concomitant statins had improved PFS compared with patients treated with niraparib only (HR = 0.75; 95% CI, 0.56–1.01; *P* = 0.027; mPFS 4.6 vs. 3.5 months; Fig. 4; Supplementary Table S5), suggesting a clinical benefit of niraparib and concomitant statin treatment versus niraparib alone. The statistical interaction in the PFS analysis between niraparib and statins could not be analyzed owing to the absence of corresponding placebo controls.

### *Post hoc* analysis of the NOVA clinical study to investigate interaction between niraparib and statins

In NOVA (14), a randomized, double-blind phase 3 trial, patients diagnosed with platinum-sensitive, high-grade serous or *BRCA*-mutated recurrent ovarian cancer were assigned to receive maintenance niraparib or placebo once daily after a minimum of four cycles of platinum-based chemotherapy. In the overall population, participants receiving niraparib had a significantly longer mPFS than those receiving placebo for all three prespecified groups (Supplementary Table S6). For the purpose of our analysis, all patients were regrouped into four groups, based on concomitant statin use. The two groups of patients taking concomitant statin treatment were (i) niraparib and statin group (*n* = 48) and (ii) placebo and statin group (*n* = 23); the other two groups (patients who were not taking concomitant statins) were (i) niraparib group (*n* = 319) and (ii) placebo group (*n* = 156).

For each efficacy population, we performed a two-sided log-rank test using randomization stratification factors to analyze PFS, which was summarized with Kaplan–Meier methods. A stratified Cox proportional hazards model was used to estimate HRs with two-sided 95% CI. Patients who received niraparib and took concomitant statin treatment had improved PFS compared with patients who received placebo with concomitant statins (HR = 0.34; 95% CI, 0.13–0.91; *P* = 0.03; mPFS 11.1 vs. 5.7 months); the benefit for niraparib versus placebo was similar among patients who did not take concomitant statins (HR = 0.38; 95% CI, 0.29–0.50; *P* < 0.001; mPFS 11.4 vs. 4.0 months; Supplementary Fig. S4; Supplementary Tables S7 and S8). The placebo with concomitant statins arm showed similar mPFS to the placebo arm, contrasting with the PRIMA analysis findings. The small number of patients in the placebo and concomitant statin arm (*n* = 23) in the NOVA trial could contribute to this inconsistency (*n* = 51 patients in the placebo and statin arm in the PRIMA trial). Thus, in this analysis of the NOVA study, although niraparib administered on a background of statin treatment had no detrimental effect, no significant interaction between niraparib and statins was observed.

In summary, longer PFS was observed in the PRIMA trial in the niraparib group with concomitant statins in comparison to niraparib alone (when each arm was compared with the respective placebo arm), whereas NOVA results showed similar mPFS in the two groups. Notably, in the PRIMA trial, we observed a significant niraparib–statin interaction (*P* = 0.005). In the QUADRA trial (no placebo arm), directional improvement of mPFS was observed, when the niraparib group with concomitant statins was compared with the niraparib alone arm (*P* = 0.027). These analyses indicate that in patients with ovarian cancer, concomitant inhibition of PARP and cholesterol biosynthesis may result in prolonged PFS.

## Discussion

To explore potential clinical benefits of combination therapy with PARPis and cholesterol-lowering drugs, we conducted a comprehensive retrospective analysis of the niraparib registrational clinical trials in ovarian cancer, QUADRA (15), PRIMA (8), and NOVA (14), and investigated the effect of statins, when administered to enrolled patients as concomitant medication for unrelated cardiovascular conditions.

In this analysis, the statin–niraparib combination showed significantly better PFS efficacy than niraparib alone in the PRIMA trial (*P* = 0.005 for niraparib–statin interaction), indicating that concomitant inhibition of PARP and cholesterol biosynthesis can be significant in patients with ovarian cancer. The mPFS was longest in the subgroup with concomitant statins, even though patients receiving placebo with concomitant statins had the worst outcome, which may be attributed to cardiovascular conditions or the older age and greater body weight among patients receiving statins. The fact that in the reported analysis, statin therapy without niraparib does not seem to have any clinical benefit on oncologic outcomes is consistent with the hypothesis that niraparib and statins could act synergistically to suppress the progression of ovarian cancer. Interestingly, our findings indicate also a possible advantage in the co-administration of niraparib and statins for ovarian cancer treatment, regardless of the HR status of the tumors. However, these are initial observations and require further investigation to substantiate the results and elucidate the underlying mechanisms, which might be different from the cannonical synthetic lethality.

In the QUADRA trial, with no placebo arm, directional improvement in mPFS was observed, when the niraparib group with concomitant statins was compared with the niraparib alone arm (*P* = 0.027). Of note, the analysis of NOVA clinical data showed a similar effect for niraparib and niraparib–statin groups (when compared with the respective placebo arm), although this may be a chance finding due to the limited number of patients with concomitant statins in that study (*n* = 71 patients vs. *n* = 105 and *n* = 143, for QUADRA and PRIMA, respectively).

Although unable to discriminate between a mechanistic and a purely associative effect, our analysis reveals a previously undescribed interaction between baseline statins, administered for cardiovascular conditions and improved clinical outcome of patients with cancer treated with niraparib. However, the different interactions observed in the analyzed trials can be confounded by several limitations of our study: (i) the retrospective design with regard to the statin–PARPi interaction and the lack of data reporting cholesterol levels of the enrolled patients, (ii) differences in the design of the PRIMA, NOVA, and QUADRA trials, including patient characteristics, (iii) the differing numbers of patients with concomitant statin across the studies. To gain better understanding of the interaction between statins and PARPis as a determinant of a multivariable analysis assessing improved response in oncologic indications, prospective clinical trials should be considered, not only driven by preexisting hypercholesterolemia and/or cardiovascular conditions. Importantly, monitoring of cholesterol levels in all patients (independently of any associated conditions) should be included to aid mechanistic understanding, also in context of potential AE. Increased cholesterol levels and association with hypercholesterolemia were reported for one PARPi, rucaparib (18, 19).

A growing body of evidence suggests altered metabolic reprogramming is a hallmark of cancer, e.g., dysregulation of cholesterol metabolism has been linked to tumor progression, metastasis, and chemoresistance in ovarian cancer (20). Research shows that cholesterol and its metabolites can promote ovarian cancer cell proliferation and survival, leading to more aggressive disease behavior (20, 21). Elevated levels of low-density lipoprotein cholesterol, specifically, have been associated with poorer progression-free and disease-specific survival in patients with ovarian cancer (22). Thus, targeting key metabolic pathways can provide new anticancer therapeutic strategies (23, 24). Certain molecular subtypes of cancer, both solid tumors as well as diffuse ones, were reported to be vulnerable to statins, FDA-approved cholesterol-lowering drugs, potent inhibitors of the rate-limiting enzyme of the mevalonate pathway, HMGCR (11). There is a significant interest in repurposing statins as anticancer agents (25), because several large retrospective population-based analyses reported that the use of statins after diagnosis can lead to better ovarian cancer–specific survival (26, 27) and some clinical studies have reported association of statins in combination with other anticancer therapies (11, 12). To the best of our knowledge, this is the first report of a *post hoc* analysis of a clinical trial showing significant interaction with PARPi.

Importantly, the evidence of the benefits of statin combinations for oncologic indications remains a subject of ongoing scientific discourse and investigation (11, 12). The majority of existing research has primarily focused on the direct effects of statins on induction of apoptosis and tumor cell killing, given the essential role of cholesterol and nonsterol isoprenoids in cellular proliferation and survival of tumor cells (28). Significant alterations of cholesterol and lipid metabolism have been specifically identified in triple-negative breast cancer (29) and breast cancer–derived brain metastases (30, 31). However, statins were also reported to affect cellular pathways beyond cholesterol biosynthesis and have pleiotropic effects such as decreased inflammation and oxidative stress, regulation of angiogenesis and osteogenesis, improvement in endothelial function (25, 32). Furthermore, akin to PARPis, statins have been reported to modulate the intratumoral immunity (33–36). Thus, in addition to their direct impact on tumor cell viability, which might necessitate the accumulation of statins at the tumor site, statins may also exert effects on other tissues and cell types, including the immune system, which could contribute to the observed synergy and improved PFS seen with the combination of statins and PARPi. Lastly, both PARP enzymes and PARPis have been reported to play a role in modulation of cholesterol and/or lipid metabolism (37), and the combination of PARPis with statins was shown to have a synergistic effect on tumor cell killing *in vitro* (13). Therefore, further investigations and a detailed molecular characterization of the tumor microenvironment are required to enhance our understanding of the potential clinical benefit of concomitant niraparib and statin treatment. This will also facilitate the identification of predictive biomarkers necessary for selecting cancer subtypes susceptible to such combination therapy.

## Supplementary Material

Table S1 Patient characteristics for PRIMA/NOVA/QUADRA – statins concomitant

Table S2 Results of analysis from the PRIMA clinical trials

Table S3 PRIMA Patient Characteristics and Baseline Demographics

Table S4 Adverse events in the different subgroups of the PRIMA study

Table S5 QUADRA patient characteristics and baseline demographics

Table S6 Results of analysis from the NOVA clinical trials

Table S7 NOVA patient characteristics and baseline demographics

Table S8 Adverse events in the different subgroups of the NOVA study

Figure S1 Retrospective analysis of PRIMA clinical trial – stratified by patient characteristics (age and weight) and statin treatment (type of statin and its dose level)

Figure S2 Retrospective analysis of PRIMA clinical trial in the HRd and HRp groups – stratified by patient characteristics (age and weight) and statin treatment (type of statin and its dose level)

Figure S3 Retrospective analysis of PRIMA clinical trial - the interaction between niraparib and statin is significant (stratified by start dose level of 200 and 300 mg)

Figure S4 Retrospective analysis of NOVA clinical trial
