# Supplementary material for: Potential Synergistic Effect between Niraparib and Statins in Ovarian Cancer Clinical Trials
Source: Cancer Res Commun. 2025 Jan 29;5(1):178–86. doi: 10.1158/2767-9764.CRC-24-0191 (PMC11775730; doi:10.1158/2767-9764.CRC-24-0191)
Supplement: Table S1 — Patient characteristics for PRIMA/NOVA/QUADRA – statins concomitant [file crc-24-0191_table_s1_suppst1.docx]

**Supplementary Table S1: Patient characteristics for PRIMA/NOVA/QUADRA –
statins concomitant**


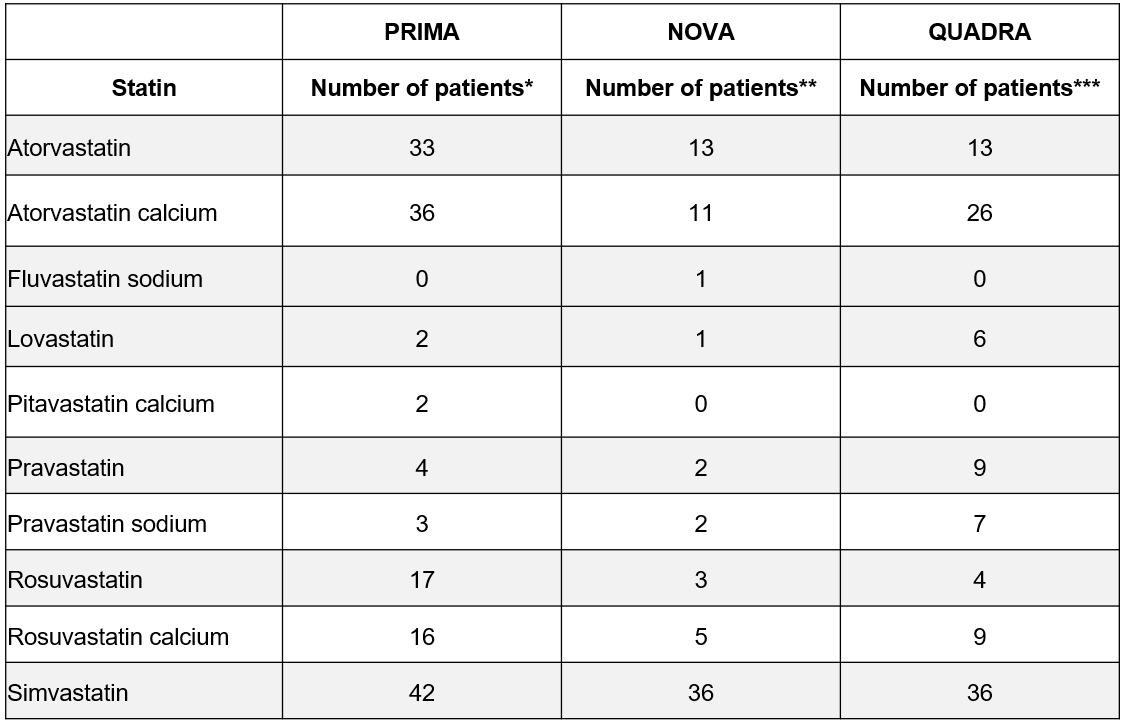


* 9 patients were taking more than one statin
** 3 patients were taking more than one statin
*** 4 patients were taking more than one statin
