# Supplementary material for: Potential Synergistic Effect between Niraparib and Statins in Ovarian Cancer Clinical Trials
Source: Cancer Res Commun. 2025 Jan 29;5(1):178–86. doi: 10.1158/2767-9764.CRC-24-0191 (PMC11775730; doi:10.1158/2767-9764.CRC-24-0191)
Supplement: Table S2 — Results of analysis from the PRIMA clinical trials [file crc-24-0191_table_s2_suppst2.docx]

**Supplementary Table S2. Results of analysis from the PRIMA clinical trials^1^**


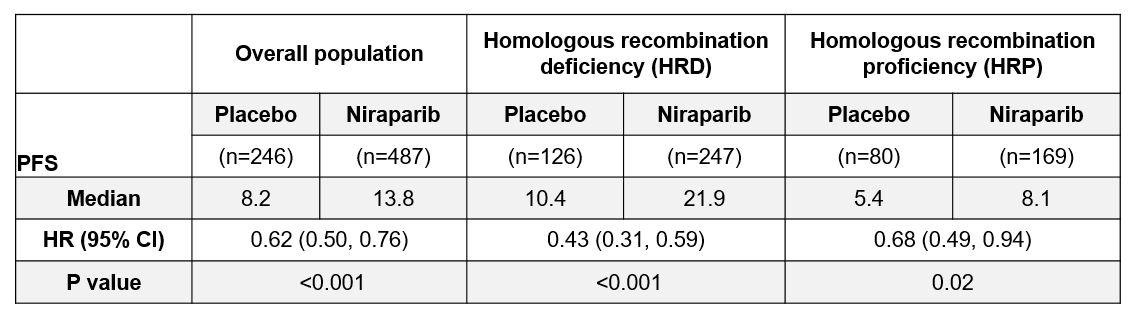


PFS, progression-free survival; HR, hazard ratio; CI, confidence interval;

^1^Gonzalez-Martin, A., et al. Niraparib in Patients with Newly Diagnosed Advanced Ovarian Cancer. N Engl J Med 381, 2391-2402 (2019)
