# Supplementary material for: Potential Synergistic Effect between Niraparib and Statins in Ovarian Cancer Clinical Trials
Source: Cancer Res Commun. 2025 Jan 29;5(1):178–86. doi: 10.1158/2767-9764.CRC-24-0191 (PMC11775730; doi:10.1158/2767-9764.CRC-24-0191)
Supplement: Table S3 — PRIMA Patient Characteristics and Baseline Demographics [file crc-24-0191_table_s3_suppst3.docx]

**Supplementary Table S3: PRIMA Patient Characteristics and Baseline Demographics**


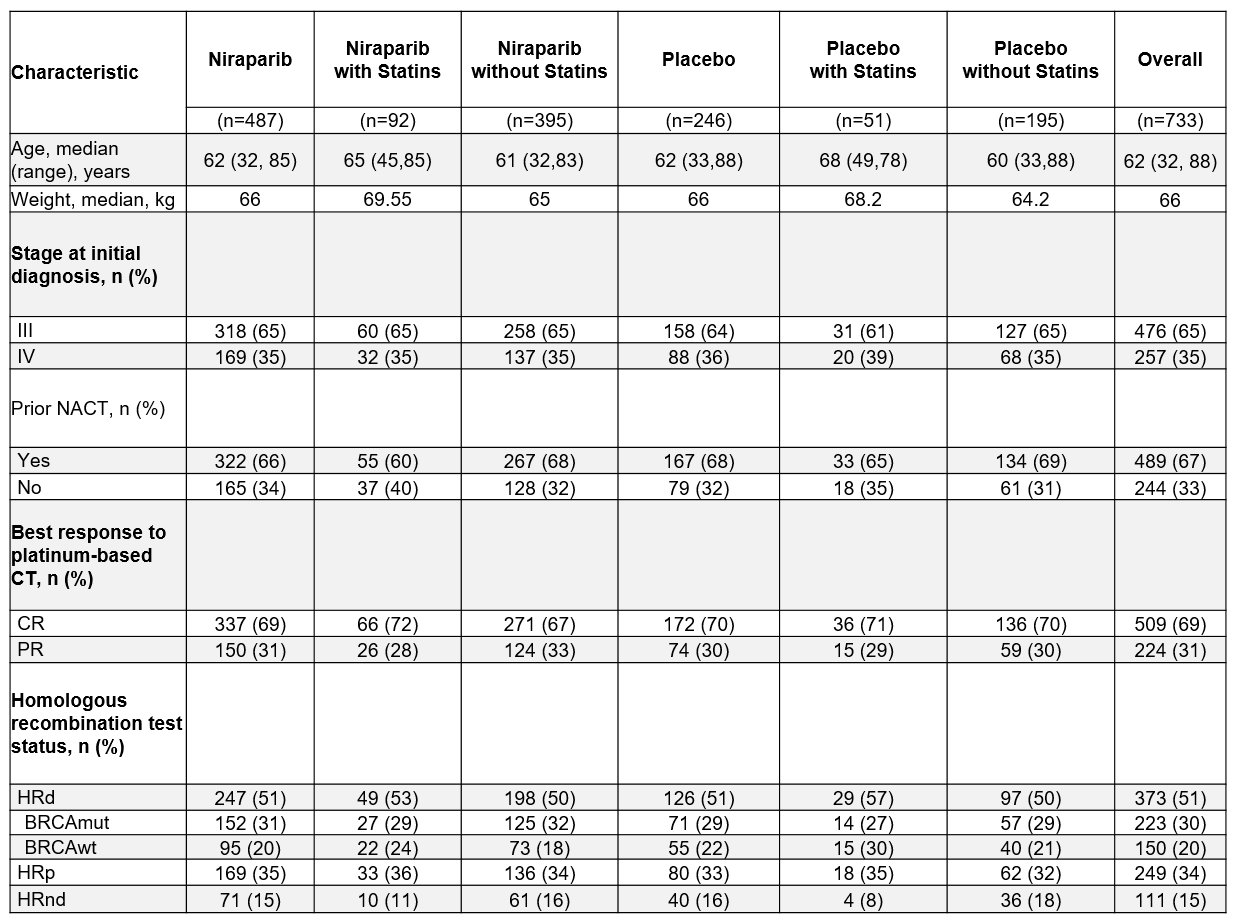


35% of patients were Stage IV, 99.6% with Stage III had residual disease post PDS, 67% received NACT, 31% achieved a PR to 1L CT, 51% had HRd tumors, 30% had BRCAmut tumors, 34% had HRp tumors

1L, first-line; CR, complete response; CT, chemotherapy; HRd, homologous recombination deficient; HRp, homologous recombination proficient; HRnd, homologous recombination not determined; mut, mutation; NACT, neoadjuvant chemotherapy; PR, partial response; wt, wild-type.
