# Supplementary figures and images for: Potential Synergistic Effect between Niraparib and Statins in Ovarian Cancer Clinical Trials
Source: Cancer Res Commun. 2025 Jan 29;5(1):178–86. doi: 10.1158/2767-9764.CRC-24-0191 (PMC11775730; doi:10.1158/2767-9764.CRC-24-0191)

**Supplementary Table S4: Adverse events in the different subgroups of the PRIMA study**


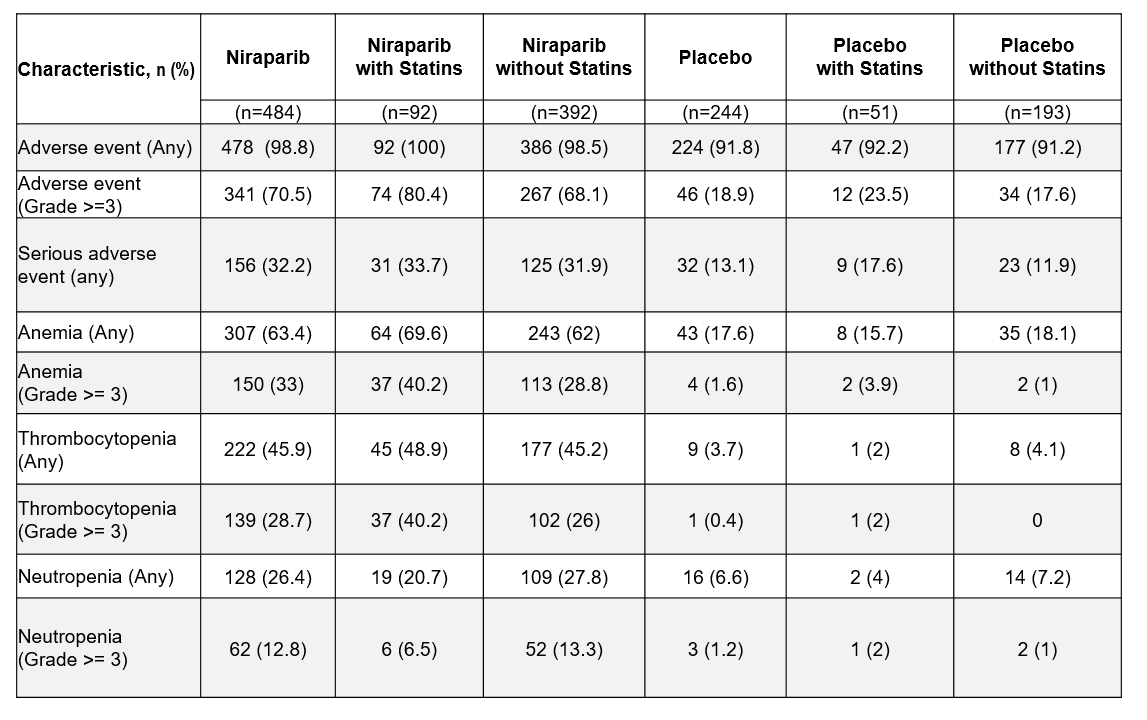

Supplement: Table S4 — Adverse events in the different subgroups of the PRIMA study [file crc-24-0191_table_s4_suppst4.docx]

**Supplementary Table S5: QUADRA patient characteristics and baseline demographics**


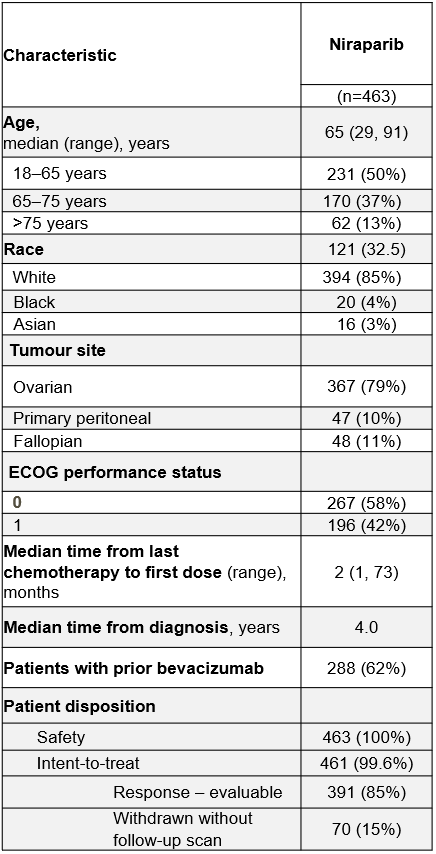

Supplement: Table S5 — QUADRA patient characteristics and baseline demographics [file crc-24-0191_table_s5_suppst5.docx]

**Supplementary Table S8: Adverse events in the different subgroups of the NOVA study**


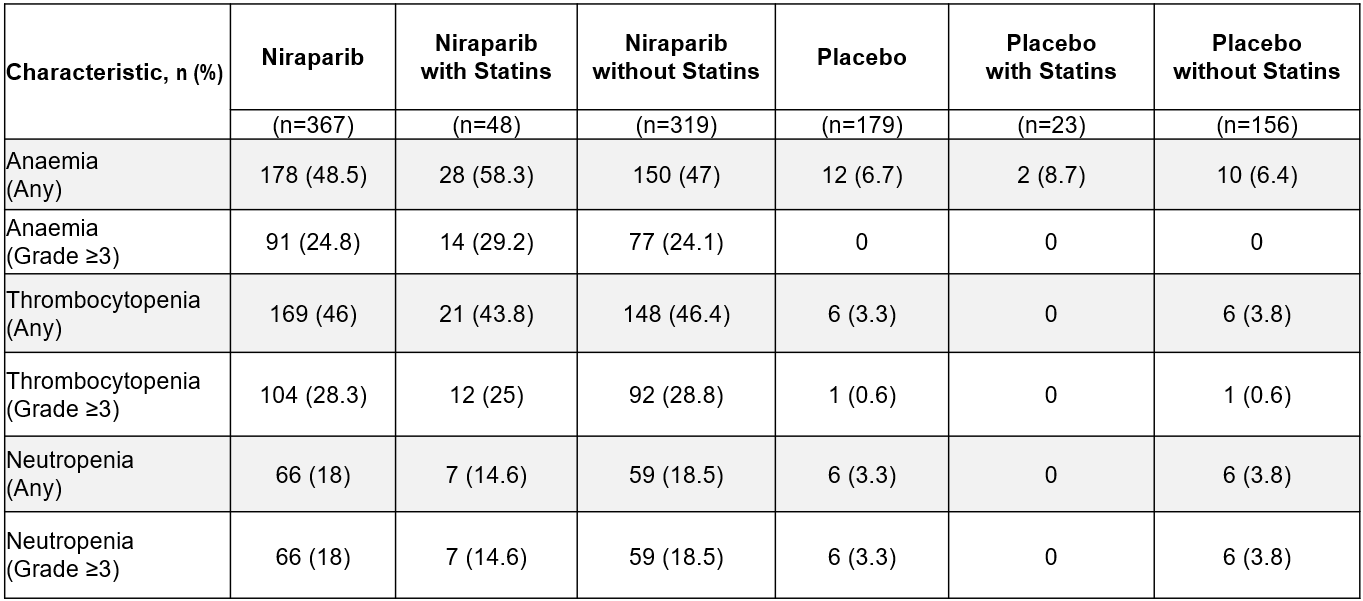

Supplement: Table S8 — Adverse events in the different subgroups of the NOVA study [file crc-24-0191_table_s8_suppst8.docx]
