# Supplementary material for: Potential Synergistic Effect between Niraparib and Statins in Ovarian Cancer Clinical Trials
Source: Cancer Res Commun. 2025 Jan 29;5(1):178–86. doi: 10.1158/2767-9764.CRC-24-0191 (PMC11775730; doi:10.1158/2767-9764.CRC-24-0191)
Supplement: Table S6 — Results of analysis from the NOVA clinical trials [file crc-24-0191_table_s6_suppst6.docx]

**Supplementary Table S6: Results of analysis from the NOVA clinical trials^1^**


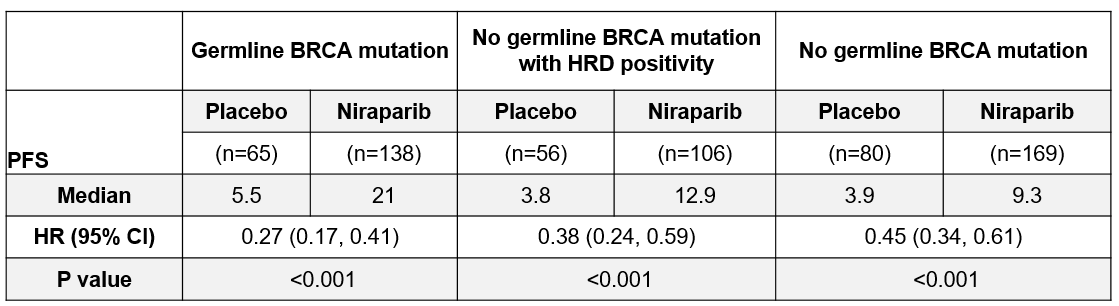


PFS, progression-free survival; HR, hazard ratio; CI, confidence interval;

^1^Mirza, M.R., et al. Niraparib Maintenance Therapy in Platinum-Sensitive, Recurrent Ovarian Cancer. N Engl J Med 375, 2154-2164 (2016)
