# Supplementary material for: Potential Synergistic Effect between Niraparib and Statins in Ovarian Cancer Clinical Trials
Source: Cancer Res Commun. 2025 Jan 29;5(1):178–86. doi: 10.1158/2767-9764.CRC-24-0191 (PMC11775730; doi:10.1158/2767-9764.CRC-24-0191)
Supplement: Table S7 — NOVA patient characteristics and baseline demographics [file crc-24-0191_table_s7_suppst7.docx]

**Supplementary Table S7: NOVA patient characteristics and baseline demographics**


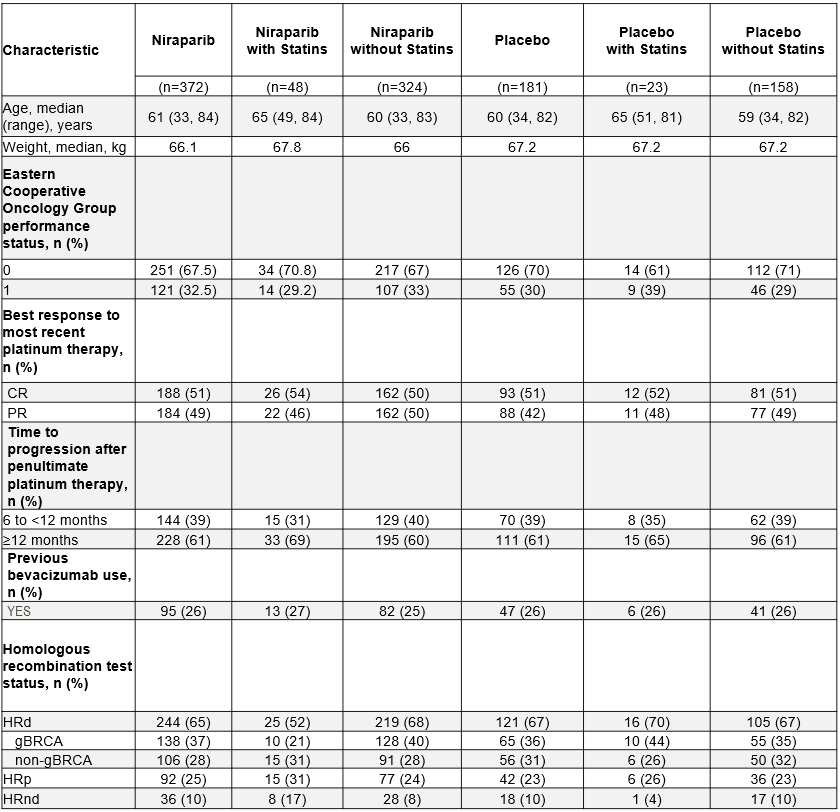


CR, complete response; gBRCA, germline BRCA; HRd, homologous recombination deficient; HRp, homologous recombination proficient; HRnd, homologous recombination not determined; PR, partial response.
