# Supplementary material for: Potential Synergistic Effect between Niraparib and Statins in Ovarian Cancer Clinical Trials
Source: Cancer Res Commun. 2025 Jan 29;5(1):178–86. doi: 10.1158/2767-9764.CRC-24-0191 (PMC11775730; doi:10.1158/2767-9764.CRC-24-0191)
Supplement: Figure S1 — Retrospective analysis of PRIMA clinical trial – stratified by patient characteristics (age and weight) and statin treatment (type of statin and its dose level) [file crc-24-0191_figure_s1_suppsf1.docx]

**Supplementary Figure S1:** Retrospective analysis of PRIMA clinical trial – stratified by patient characteristics (age and weight) and statin treatment (type of statin and its dose level)


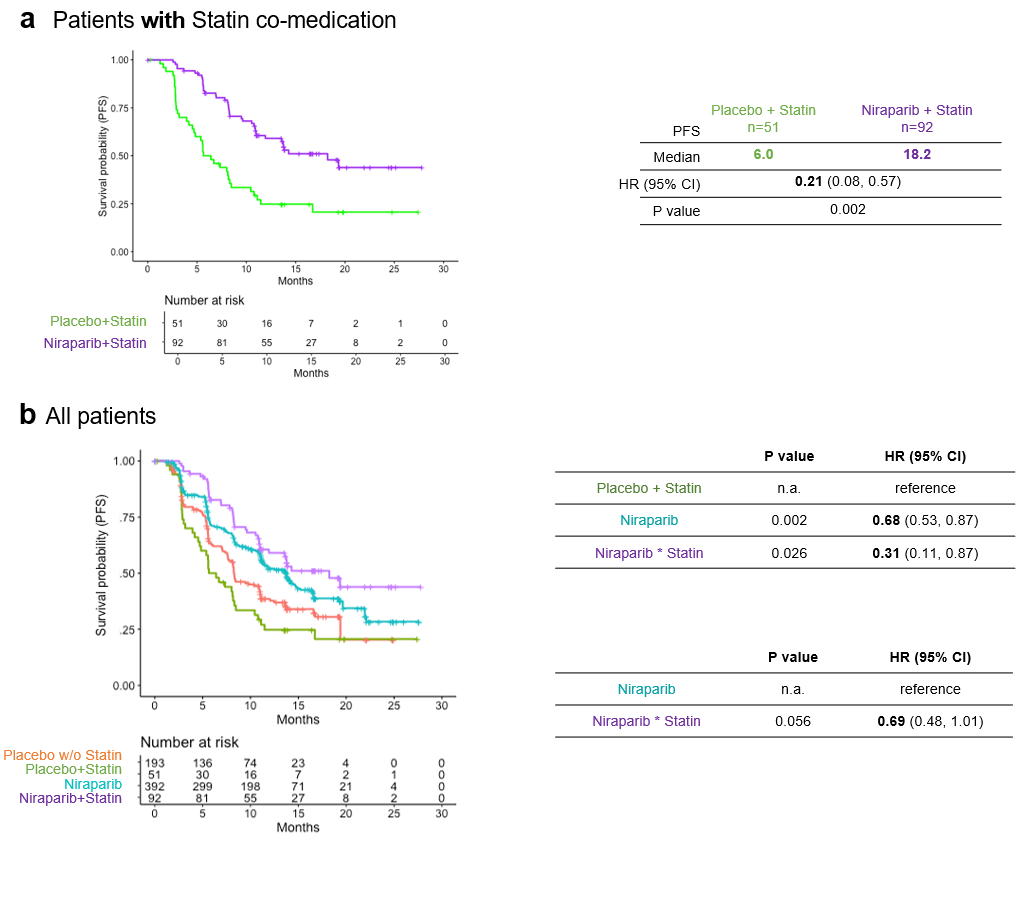


**a**, Retrospective analysis of the PRIMA trial – Kaplan–Meier estimation of progression-free survival (PFS) comparing patients treated with niraparib (n=92) and placebo (n=51) in the cohort with statin concomitant; stratified analysis with 7 randomization factors (response to first platinum, homologous recombination deficiency (HRd) status, and neoadjuvant usage, patient age, patient weight, type of statin and its dose level); mPFS, median progression-free survival; HR, hazard ratio, and log-rank test P value are reported. **b**, Kaplan–Meier estimation of PFS comparing three arms (the placebo with statins was the reference arm for comparison to the other 2 arms: niraparib, niraparib+statin) as well as two arms (niraparib was the reference arm for comparison to niraparib+statin arm).
