# Supplementary material for: Potential Synergistic Effect between Niraparib and Statins in Ovarian Cancer Clinical Trials
Source: Cancer Res Commun. 2025 Jan 29;5(1):178–86. doi: 10.1158/2767-9764.CRC-24-0191 (PMC11775730; doi:10.1158/2767-9764.CRC-24-0191)
Supplement: Figure S3 — Retrospective analysis of PRIMA clinical trial - the interaction between niraparib and statin is significant (stratified by start dose level of 200 and 300 mg) [file crc-24-0191_figure_s3_suppsf3.docx]

**Supplementary Figure S3:** Retrospective analysis of PRIMA clinical trial stratified by start dose level of 200 mg and 300 mg


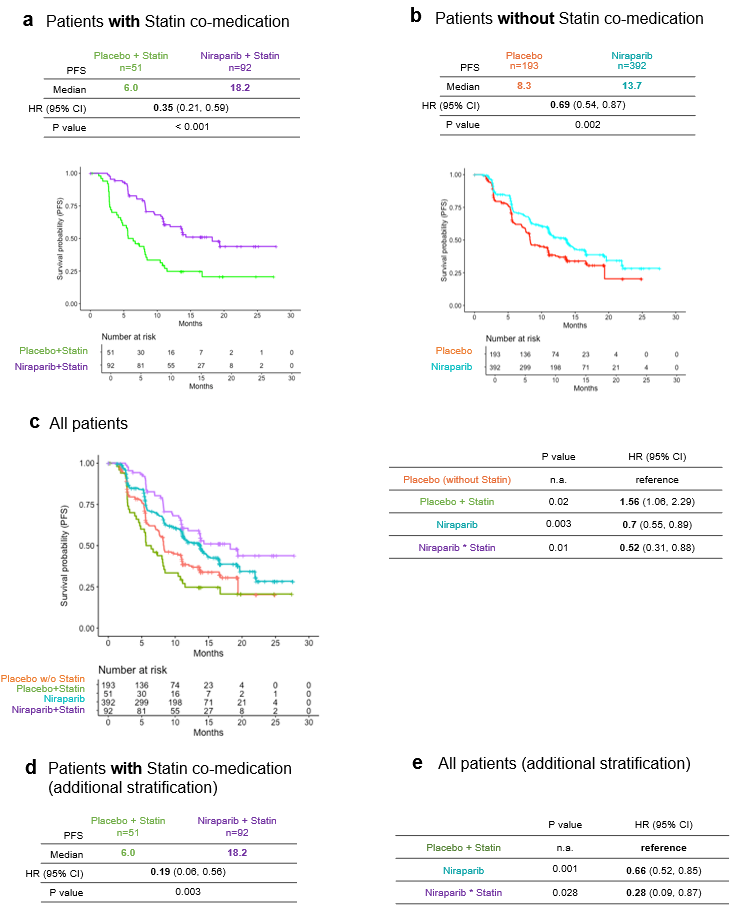


**a**, Retrospective analysis of the PRIMA trail - Kaplan–Meier estimation of progression-free survival (PFS) comparing patients treated with niraparib (n=92) and placebo (n=51) in the cohort with statin concomitant; stratified analysis (4 randomization factors: response to first platinum, homologous recombination deficiency (HRd) status, and neoadjuvant usage and niraparib start dose); mPFS, median progression-free survival; HR, hazard ratio, and log-rank test P value are reported. **b**, Kaplan–Meier estimation of PFS comparing patients treated with niraparib (n=392) and placebo (n=193) in the cohort without statin concomitant. **c**, Kaplan–Meier estimation of PFS comparing all four arms (the placebo without statins was the reference arm for comparison to the other 3 arms: niraparib, niraparib+statin, placebo+statin). Statin and Niraparib showed synergistic combination effect (significant interaction) in this retrospective analysis. **d**, Kaplan–Meier estimation of progression-free survival (PFS) comparing patients treated with niraparib (n=92) and placebo (n=51) in the cohort with statin concomitant; stratified analysis with 8 randomization factors (response to first platinum, HRd status, and neoadjuvant usage, patient age, patient weight, type of statin and its dose level and niraparib start dose). **e**, Kaplan–Meier estimation of PFS comparing the three arms in the 8 stratification factor analysis as described in d (the placebo with statins was the reference arm for comparison to the other 2 arms: niraparib, niraparib+statin). Statin and niraparib showed synergistic combination effect (significant interaction) in this retrospective analysis.
