# Supplementary material for: Potential Synergistic Effect between Niraparib and Statins in Ovarian Cancer Clinical Trials
Source: Cancer Res Commun. 2025 Jan 29;5(1):178–86. doi: 10.1158/2767-9764.CRC-24-0191 (PMC11775730; doi:10.1158/2767-9764.CRC-24-0191)
Supplement: Figure S4 — Retrospective analysis of NOVA clinical trial [file crc-24-0191_figure_s4_suppsf4.docx]

**Supplementary Figure S4:** Retrospective analysis of NOVA clinical trial


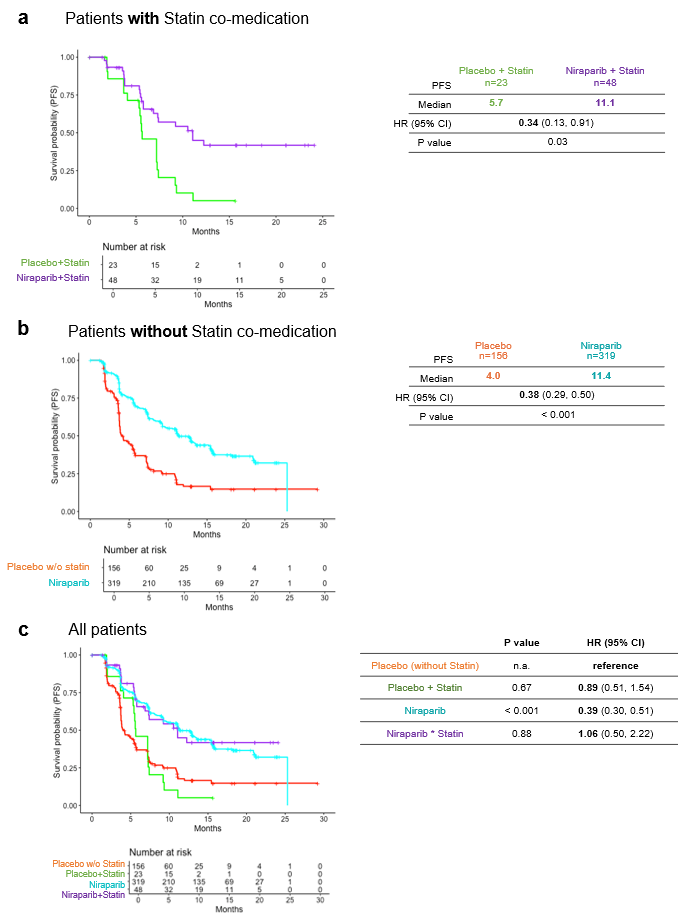


**a**, Retrospective analysis of the NOVA trial - Kaplan–Meier estimation of progression-free survival (PFS) comparing patients treated with niraparib (n=48) and placebo (n=23) in the cohort with statin concomitant; stratified analysis with 3 randomization factors (the best response during the last platinum, the use of bevacizumab in conjunction with the penultimate or last platinum regimen, and time to progression after completion of the penultimate platinum regimen (6 to <12 months vs ≥12 months); mPFS, median progression-free survival; HR, hazard ratio, and log-rank test P value are reported. **b**, Kaplan–Meier estimation of PFS comparing patients treated with niraparib (n=319) and placebo (n=156) in the cohort without statin concomitant. **c**, Kaplan–Meier estimation of PFS comparing all four arms (the placebo without statins was the reference arm for comparison to the other 3 arms: niraparib, niraparib+statin, placebo+statin).
